# Plague in India

**Published:** 1900-03-17

**Authors:** 


### PLAGUE IN INDIA.

The interest which we all take in South African affairs is, perhaps, responsible for the fact that one hears so little about the plague in India, notwithstanding the extraordinary development and extension of the epidemic in that country during recent weeks. The number of cases which are reported in Bombay is far greater now than it was even when, a few years ago, the whole world was full of excited interest in regard to the daily progress of the disease. When we understand that the death-rate in this unfortunate city has lately been more than five times as high as what may be termed its normal level, which itself is a high one, and when we find that within the past few weeks the people there have been dying at a rate which, if continued, would mean that a sixth of the whole population would die within the year, we can, perhaps, grasp the seriousness of the position. Meanwhile, the disease is spreading in other parts of India, and the hostility aroused by the not very judicious attempts made in previous years to enforce segregation and other obnoxious preventive measures has led to a *laisser faire* attitude on the part of the officials which does not augur well for the future. The population question in India thus threatens to solve itself by aid of plague and famine, much as it used to do a couple of hundred years ago before the introduction of Western ideas. It is a somewhat savage method, but effectual.

